# Craniocervical posture in patients with skeletal malocclusion and its correlation with craniofacial morphology during different growth periods

**DOI:** 10.1038/s41598-024-55840-w

**Published:** 2024-03-04

**Authors:** Houli Peng, Weihan Liu, Lanxin Yang, Pingping Yan, Wenjie Zhong, Xiang Gao, Jinlin Song

**Affiliations:** 1 College of Stomatology, Chongqing Medical University, Chongqing, 401147 China; 2 Chongqing Key Laboratory of Oral Diseases and Biomedical Sciences, Chongqing, 401147 China; 3 Chongqing Municipal Key Laboratory of Oral Biomedical Engineering of Higher Education, Chongqing, 401147 China; 4 Department of Orthodonticsrthodontics, Chongqing University Three Gorges Hospital, Chongqing, 404000 China

**Keywords:** Craniofacial morphology, Sagittal skeletal discrepancy, Head and cervical posture, Facial growth, Pubertal growth, Dentistry, Dental public health, Occlusion, Orthodontics, Preventive dentistry

## Abstract

The association between craniocervical posture and craniofacial structures in the various sagittal skeletal malocclusion during different growth stages has been the focus of intense interest in fields of orthodontics, but it has not been conclusively demonstrated. Thus, this study aimed to investigate the association between craniofacial morphology and craniocervical posture in patients with sagittal skeletal malocclusion during different growth periods. A total of 150 from a large pool of cephalograms qualified for the inclusion and exclusion were evaluated and classified into three groups according to the Cervical Vertebral Maturation (CVM) by examining the morphological modifications of the second through fourth cervical vertebrae, each group consisted of 50 cephalograms. In each growth period, for the comparison of head and cervical posture differences among various skeletal classes, the radiographs were further subdivided into skeletal Class I (0° < ANB < 5°, n = 16), skeletal Class II (ANB ≥ 5°, n = 18), and skeletal Class III (0° ≤ ANB, n = 16) on the basis of their ANB angle. There was no significant difference in gender (*P* > 0.05). Some variables were found to be significant during pubertal growth and later in patients with sagittal skeletal malocclusion (*P* < 0.05). Most indicators describing craniocervical posture were largest in skeletal Class II and smallest in skeletal Class III during the peak growth periods and later. Cervical inclination variables were greater in skeletal Class III than in skeletal Class II. Variables of craniofacial morphology and craniocervical posture are more correlated during the pubertal growth period and later in patients with sagittal skeletal malocclusion. A tendency is an indication of the close interrelationship that a more extended head was in skeletal Class II while a flexed head was in skeletal Class III. Nevertheless, with the considerations of some limitations involved in this study, further longitudinal studies with large samples are required to elucidate the relationship clearly.

## Introduction

Malocclusion is defined as a deformity of the dentition and the craniofacial skeleton deriving from genetic and environmental factors, which is considered as the third priority of oral conditions followed by caries and periodontal diseases, having an impact on the physical and mental health^1^. At present, it has gradually become one of the motivations for people to seek orthodontic treatments. It can not only affect esthetics of the face, but can also contribute to functional issues, such as difficulty in mastication, phonetics, and even mental health of patients^2,3^. Concerning the mechanisms of occlusion alterations, although the effects of these etiological factors have not been fully understood, a growing number of researchers have attached more importance to the influence of environmental factors on the occurrence of malocclusion^4,5^. The stomatognathic system is possibly implicated in the postural system via the various muscular groups and functions as an interconnected and coordinated apparatus involved in dental occlusions, temporomandibular joints and related muscles. Any abnormalities or variations in this system can negatively influence the behavior of other systems disrupting postural stability and potentially leading to cranio-cervical-mandibular disorders^6,7^.

Craniocervical posture, refers to alignment of head upon the cervical vertebrae in space, is a biomechanical position of muscular and skeletal balance. It was explained by a close morphological and functional connection of cervical spine with craniofacial structures, which plays a role of a transitional zone of head and cervical region^8^. Optimal cervical lordosis is an important physiological curve for maintaining the mechanical stability and function of the cervical spine^9,10^. In recent years, a growing number of investigations support^11–15^ a developmental link between malocclusion and improper head and cervical posture, which is primarily explained by the “soft tissue stretching hypothesis”, stating a head extension may predispose individuals to a passive stretching of soft tissues generating stress on cervical structures by increasing the amount of antigravity load, which also restrains the normal forward growth of the facial structures^16^. Previous investigations have demonstrated significant correlations linking the poor cervical posture, temporomandibular joint disorders, nasopharyngeal airway obstruction, which is in association with to the development of Class II malocclusion^17–20^. It was also found that an increased vertical facial development, a steeper inclination of the mandible and a very high probability of skeletal class II malocclusion with a convex profile in subjects were accompanied by poor craniocervical posture^16,21^. However, several studies have rejected the relationship linking malocclusion and craniocervical posture, due to a variety of possible factors such as the limits of age, gender, race, the small samples examined, and poor-quality designs used^22,23^.

Although actively debated, a consensus has not yet been reached on this matter, various studies in the literature have focused on the impact of age and gender on the correlation^24–27^. Others^22,26,28,29^ proposed that caution in the interpretation of their data due to the limitations of their study, including limited demographic information, small sample size and poor methodological quality. Mounting evidence^26,30,31^ has primarily focused on specific age groups, making it challenging to accurately evaluate the variations in cervical spine posture among different growth stages. A study conducted by Bernal et al.^26^ illustrated that craniocervical postural variables were higher in boys than girls. Gender, as a potential etiopathologic factor, could affect the association between malocclusion and head and cervical position. D’Attilio et al.^32^ stated that no additional evaluations have been made by age or gender due to the small number of subjects. However, the influence of this factor has not been consistently supported by other studies^24,30^. To reduce heterogeneity, accounting for gender and age is recommended by some researchers^33,34^. Therefore, taking into consideration that interactive and variable nature of individual growth and development, it is crucial for clinicians to investigate the relationship between occlusion and craniocervical posture at different stages of growth and development.

Despite the extensive evidence of anatomical and physiological association between malocclusion and cervical disorders^7,9,35,36^, there is no consensus on this topic about the effects of head and neck posture on maxillofacial development in patients with sagittal skeletal malocclusion throughout all the growth stages. Therefore, the objective of the present study was to investigate whether an association exists between position of the head and cervical spine, and craniofacial morphology in patients with different sagittal skeletal jaw relations during different stages of growth, clarifying the relationship between the postural variables and malocclusion may be considered an important element of orthodontic diagnosis and treatment for clinicians.

## Materials and methods

### Sample design and setting

This study is a cross-sectional study, the test level α = 0.05 was set up, the test efficacy of 1 − β = 0.09, the standard deviation σ is expected to be 4 through literature, the allowed error δ is 0.75, the Z (1 − α/2) = 1.96. According to the formula n = (Z_1−α/2_*σ/δ)^2^, the minimum total sample size was calculated (n = 109). Finally, a sample of 150 individuals were included in the present study. To reduce heterogeneity brought about by gender differences, men and women were divided equally. The study was conducted with the pretreatment lateral cephalometric radiographs, a total of 225 patients who had attended the department of orthodontics for seeking treatment were randomly selected from the record archives. 75 subjects were not included according to the inclusion and exclusion criteria. Finally, a total of 150 (75 females and 75 males, aged 7–18 years) were selected on the basis of the following inclusion criteria. The study was reviewed and approved by the local ethics committee. Informed consent was obtained from the subjects and/or their parents.

#### Inclusion and exclusion criteria

Subjects were included if they had: (1) Sagittal skeletal malocclusion; (2) Lateral cephalometric radiographs taken in the natural head position (NHP) and at least four clear cervical spine shapes; (3) A Chinese ethnic origin; (4) No history of orthodontic treatment or orthognathic surgery. Subjects were excluded if they had: (1) Deleterious oral habits such as oral breathing or chewing side preference; (2) A history of severe vertical or horizontal development collapse; (3) Neurological and respiratory diseases;

(4) Potential craniomaxillofacial disorders such as temporomandibular joint disorders or cleft lip and palate; (5) Family history.

### Lateral cephalometric radiographs

Cephalograms were routinely obtained with ProMax (Planmeca, Helsinki, Finland) in the NHP. Exposure was operated at 80 kV, 10 mv. The NHP of the patient was determined by positioning the subjects in a standing, self-balanced position in which they felt comfortable and relaxed. Evidence^37,38^ has shown excellent stability for 5 or even 15 years after the initial radiograph. Nine craniofacial morphology-associated variables of sagittal skeletal malocclusion and nine variables representing craniocervical, craniovertical, and cervicohorizontal and cervical curvature angles^16,30,32,39–41^ were measured using Myorthox measurement tool.

**Figure 1:**
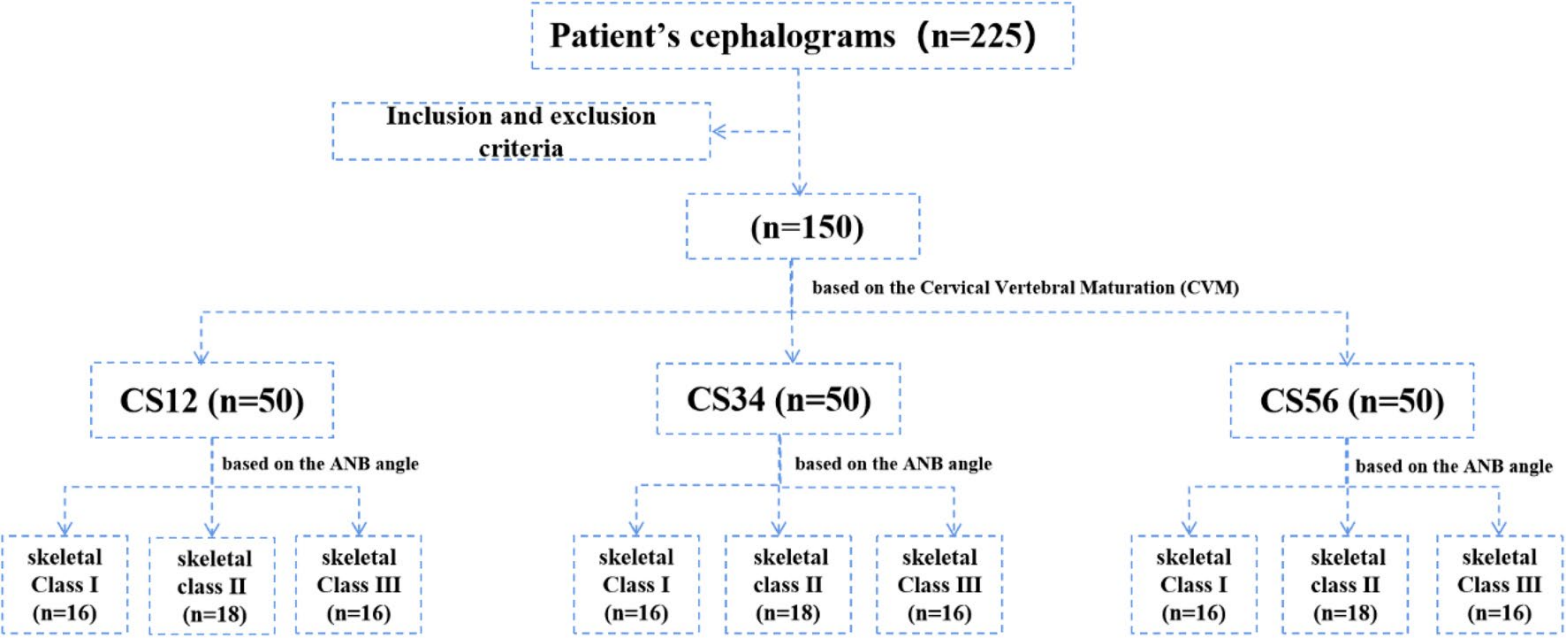
The flow chart of the sample stratification.

The cephalograms were classified into three groups according to the CVM method^42^, CS12 group (the pre-peak stage, n = 50), CS34 group (the peak growth, n = 50), and CS56 group (the post-peak stage, n = 50), representing different growth and development stages^43^ with an equal distribution of men and women by the two investigators (HP and LY). These radiographs were further subdivided into skeletal Class I (0° < ANB < 5°, n = 16, normal facial pattern without sagittal skeletal discrepancy), skeletal Class II (ANB ≥ 5°, n = 18, a convex profile showing a marked protruded maxilla or retruded mandible or a combination of both), and skeletal Class III (0° ≤ ANB, n = 16, a concave profile showing marked maxillary retrusion or mandibular protrusion or a combination of both) based on the ANB angle in each growth period, the profiles of skeletal class II and skeletal class III malocclusions are shown in Fig. S1, the flow chart of the sample stratification is shown in the Fig. 1.

### Variables and data measurement

**Figure 2:**
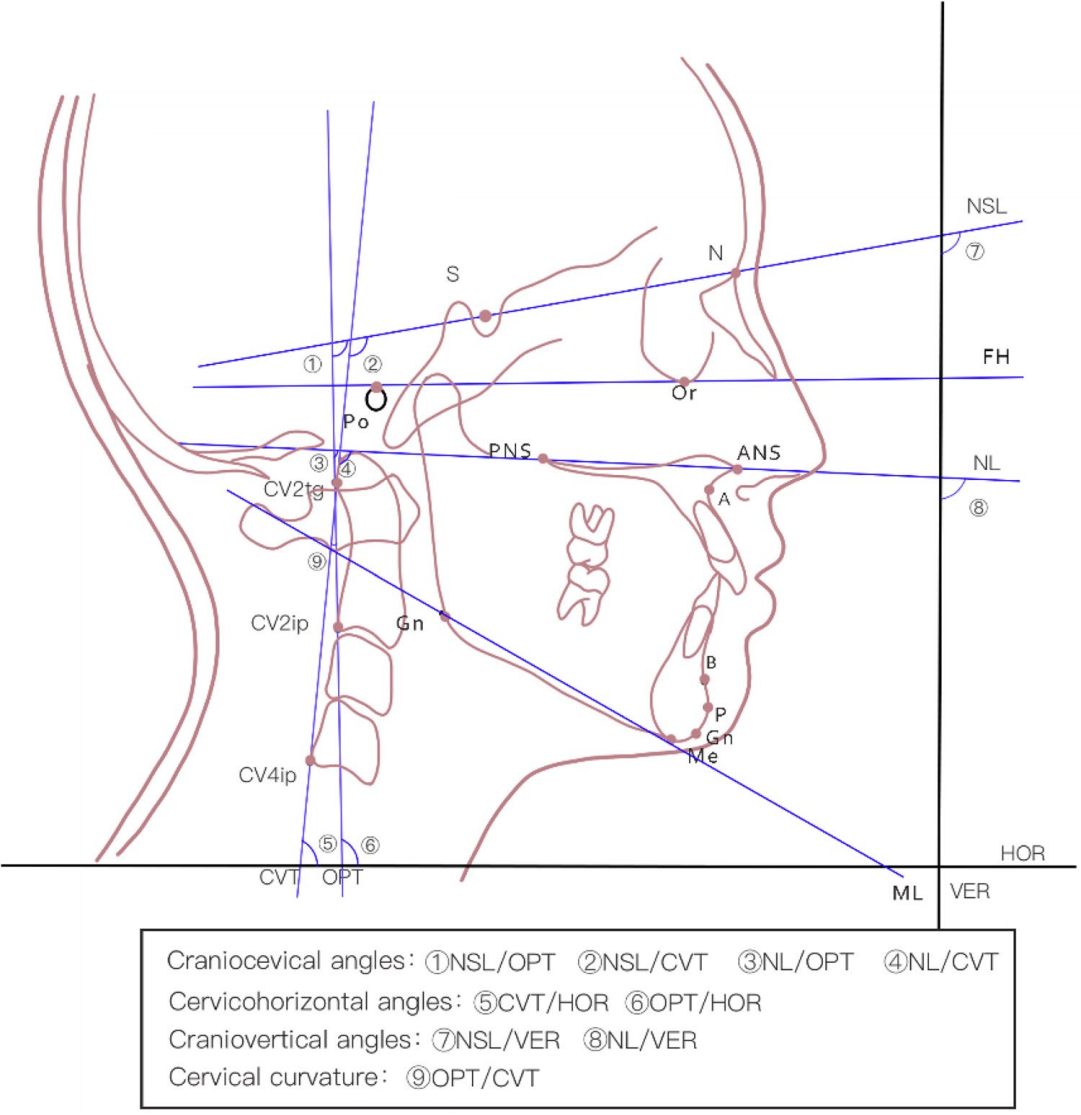
Head and cervical posture variables on the Cephalograms.

Variables associated with craniofacial morphology such as SNA, SNB, ANB, FH/ML, NSL/NL, NSL/ML, NA-PA, NP-FH, Y axis in patients with sagittal skeletal malocclusion were measured on the cephalograms. Craniocervical posture is mainly determined by the angle formed by Odontoid Process Tangent (OPT) and Nasion-Sella line (NSL) (NSL/OPT), the angle between Nasal line (NL) and OPT (NL/OPT), the angle between NSL and Cervical Vertebra Tangent (CVT) (NSL/CVT), and the angle between NL and CVT (NL/CVT). Cervical inclination was assessed using cervicohorizontal angles, such as the angle formed by OPT and horizontal line (HOR) (OPT/HOR), and the angle between CVT and HOR (CVT/HOR). Meanwhile, head position was represented using the craniovertical angles, such as the angle formed by NSL and VER (NSL/VER), and the angle between NL and VER (NL/VER). Cervical curvature was determined by measuring the angle formed by OPT and CVT (OPT/CVT). The definitions of reference points and planes are shown in Table S1, reference variables are described in Table 1, detailed landmarks and measurement items on the Cephalograms are shown in Fig. 2.

**Table 1:** Description of reference variables used for cephalometric analysis.

Reference variables	Description	Characterization
Craniofacial morphology variables		
SNA	Sella-Nasion- A angle	The prognathism of the maxilla to the cranial base^32^
SNB	Sella-Nasion-B angle	The prognathism of the mandible to the cranial base^32^
ANB	A-Nasion- B angle	Difference between SNA and SNB, which determines anterioposterior relationship of the maxillary and mandibular bases^32,40^
FH/ML	Angle between FH and ML	Frankfort mandibular plane angle^30^
NSL/ML	Angle between NSL and ML	Mandibular plane angle^30^
NSL/NL	Angle between NSL and NL	The maxilla to cranial base
NA-PA	Angle between NA and PA	Reflection of the protrusion of the maxilla
NP-FH	Angle between NP and FH	Reflection of the projection of the mandibular
Y axis	Angle between SGn and FH	Reflection of projection of the chin and the direction of facial growth
Head and cervical postural variables		
NSL/OPT	Angle between NSL and OPT^30,44^	Craniocevical angles
NSL/CVT	Angle between NSL and CVT^30,44^	
NL/OPT	Angle between NL and OPT^30,44^	
NL/CVT	Angle between NL and CVT^30,44^	
CVT/HOR	Angle between CVT and HOR^30,44^	Cervicohorizontal angles
OPT/HOR	Angle between OPT and HOR^30,44^	
NSL/VER	Angle between NSL and VER^30,44^	Craniovertical angles
NL/VER	Angle between NL and VER^30,44^	
OPT/CVT	Angle between odontoid process tangent and cervical vertebra tangent^30,44^	Cervical curvature angle

### Measurement error

Analysis of internal consistency, the intraclass correlation coefficients (ICC) were calculated for both intra- and inter-examiner concordance. An ICC value higher than 0.75 are indicative of good reliability^45^. This involved comparing the CVM groupings by two different reviewers (HP and LY) as well as analyzing the measurements taken by the same investigator (HP) after a 2-week interval.

### Statistical analysis

Descriptive variables were conducted using the Statistical Package for Social Sciences (SPSS), version 26.0 (SPSS Inc, Chicago, IL, USA). The quantitative postural variables were calculated as the mean (M) and standard deviation (SD). Pearson’s analysis was used to evaluate the correlation between craniocervical posture and craniofacial morphology in patients with sagittal skeletal malocclusion during different growth periods, and assess the potential impact of growth by comparing the correlation coefficients in various growth periods. An independent sample t-test was employed to investigate the potential influence of gender on the correlation between craniocervical posture and craniofacial morphology in sagittal skeletal malocclusion. A one-way analysis of variances (ANOVA) was used to examine and compare the intergroup differences in cervical posture across different skeletal classes during the same growth period. Significance was set at *p* ≤ 0.05.

### Ethical approval and informed consent

The study was conducted in accordance with the Declaration of Helsinki, and approved by the Ethics Committee of College of Stomatology, Chongqing Medical University (NO: 2022 LSNo.088) and Chongqing University Three Gorges Hospital (No.39 2020). The informed consent was obtained from all subjects or their parents involved in this study.

## Results

The ICC values for measurement items were determined to be higher than 0.9 for both intra- and inter-examiner assessments, suggesting a strong level of repeatability and reproducibility. Almost all values fit to a normal distribution. No postural variables were related to gender (Table 2) (*P* > 0.05). Significant differences were observed in most variables, such as the correlations between ANB, SNB, FH/ML, Y axis, NA/PA, NP/FH and NSL/VER, NSL/OPT, NL/OPT, NSL/CVT, NL/CVT, OPT/HOR, CVT/HOR during the peak and post-peak growth periods respectively. However, the correlation coefficients varied from low to moderate for most variables (Tables 3, 4 and 5).

**Table 2:** Gender differences among variables.

Variables	Male (M ± SD)	Female (M ± SD)	*P*
ANB	2.41 ± 3.87	2.53 ± 4.24	0.855
SNA	80.25 ± 4.41	81.16 ± 5.32	0.257
SNB	77.84 ± 4.56	78.62 ± 5.57	0.345
NSL/NL	10.24 ± 3.91	10.34 ± 3.88	0.873
NSL/ML	34.99 ± 6.19	36.50 ± 6.40	0.144
FH/ML	26.76 ± 6.28	27.40 ± 5.58	0.510
NA/PA	3.23 ± 8.62	4.74 ± 8.94	0.296
NP/FH	86.23 ± 4.77	85.93 ± 4.83	0.706
Y axis	62.58 ± 4.03	62.55 ± 4.24	0.964
NSL/VER	97.24 ± 7.66	95.70 ± 7.80	0.222
NL/VER	87.83 ± 5.98	87.95 ± 3.85	0.883
NSL/OPT	98.69 ± 8.91	100.06 ± 9.21	0.356
NL/OPT	88.43 ± 8.96	91.00 ± 8.84	0.079
NSL/CVT	101.45 ± 8.18	102.89 ± 8.68	0.297
NL/CVT	91.46 ± 8.20	94.07 ± 8.22	0.054
OPT/HOR	89.80 ± 8.02	87.75 ± 7.65	0.111
CVT/HOR	86.73 ± 7.31	84.40 ± 7.56	0.056
OPT/CVT	3.06 ± 3.34	3.76 ± 3.49	0.211

**Table 3:** Correlation analysis during the pre-peak growth period.

	Variables	NSL/VER	NL/VER	NSL/OPT	NL/OPT	NSL/CVT	NL/CVT	OPT/HOR	CVT/HOR	OPT/CVT
CS 1–2 group	ANB	0.285*	0.165	0.155	0.237	0.074	0.152	− 0.299*	− 0.175	− 0.183
	SNA	− 0.086	0.061	− 0.211	0.168	− 0.354*	− 0.004	− 0.148	0.015	− 0.093
	SNB	− 0.269	− 0.036	− 0.325*	0.037	− 0.431**	− 0.097	0.023	0.123	0.012
	NSL/NL	0.156	− 0.406**	0.092	− 0.345*	0.159	− 0.289*	0.157	0.032	0.155
	NSL/ML	0.336*	− 0.080	0.267	− 0.124	0.391**	0.068	0.058	− 0.085	0.143
	FH/ML	0.295*	0.053	0.026	0.009	− 0.046	0.047	− 0.060	− 0.017	− 0.018
	NA/PA	0.173	0.087	0.105	0.101	− 0.002	− 0.054	− 0.141	− 0.010	− 0.216
	NP/FH	− 0.380**	− 0.273	− 0.080	− 0.171	− 0.032	− 0.169	0.188	0.145	0.045
	Y axis	0.293*	0.195	0.065	0.182	− 0.047	0.136	− 0.249	− 0.135	− 0.160

*: *P* < 0.05, **: *P* < 0.01.

**Table 4:** Correlation analysis at the peak growth period.

	Variables	NSL/VER	NL/VER	NSL/OPT	NL/OPT	NSL/CVT	NL/CVT	OPT/HOR	CVT/HOR	OPT/CVT
CS 3–4 group	ANB	0.389**	0.395**	0.399**	0.419**	0.421**	0.402**	− 0.267	− 0.288*	0.014
	SNA	− 0.212	0.088	− 0.210	− 0.017	− 0.177	0.034	0.078	0.033	0.103
	SNB	− 0.574**	− 0.312*	− 0.583**	− 0.429**	− 0.575**	− 0.367**	0.333*	0.314*	0.078
	NSL/NL	0.241	− 0.251	0.064	− 0.183	0.067	− 0.243	0.097	0.126	− 0.050
	NSL/ML	0.161	− 0.006	0.251	0.163	0.343*	0.210	− 0.231	− 0.256	0.024
	FH/ML	0.209	0.161	0.391**	0.346*	0.419**	0.336*	− 0.327*	− 0.390**	0.094
	NA/PA	0.351*	0.320*	0.333*	0.330*	0.297*	0.263	− 0.186	− 0.156	− 0.083
	NP/FH	− 0.327*	− 0.280	− 0.504**	− 0.481**	− 0.550**	− 0.477**	0.400**	0.482**	− 0.125
	Y axis	0.334*	0.273	0.554**	0.502**	0.573**	0.483**	− 0.458**	− 0.487**	0.008

*: *P* < 0.05, **: *P* < 0.01.

**Table 5:** Correlation analysis among the post-peak growth period.

	Variables	NSL/VER	NL/VER	NSL/OPT	NL/OPT	NSL/CVT	NL/CVT	OPT/HOR	CVT/HOR	OPT/CVT
CS5-6 group	ANB	0.283*	0.483**	0.269	0.345*	0.386**	0.472**	− 0.050	− 0.218	0.320*
	SNA	− 0.508**	− 0.176	− 0.409**	− 0.179	− 0.306*	− 0.066	0.059	− 0.081	0.302*
	SNB	− 0.676**	− 0.548**	− 0.579**	− 0.438**	− 0.583**	− 0.443**	0.093	0.107	0.004
	NSL/NL	0.426**	− 0.186	0.449**	0.003	0.354*	− 0.124	− 0.163	− 0.016	− 0.333*
	NSL/ML	0.291*	0.128	0.176	0.063	0.157	0.082	0.028	0.033	0.003
	FH/ML	0.013	0.052	− 0.085	− 0.052	− 0.040	0.011	0.099	0.042	0.146
	NA/PA	0.302*	0.413**	0.281*	0.298*	0.383**	0.409**	− 0.042	− 0.190	0.282*
	NP/FH	− 0.422**	− 0.512**	− 0.334*	− 0.333*	− 0.412**	− 0.432**	0.011	0.147	− 0.262
	Y axis	0.341*	0.386**	0.248	0.239	0.335*	0.310*	0.011	− 0.095	0.216

*: *P* < 0.05, **: *P* < 0.01.

### Impact of growth period on the relationship between craniofacial morphology and craniocervical posture in sagittal skeletal malocclusion

#### Correlation analysis of variables at the pre-peak growth period

ANB was positively correlated with most variables, NSL/VER, NL/VER, NSL/OPT, NL/OPT, NSL/CVT, NL/CVT, and inversely correlated with OPT/HOR, CVT/HOR, OPT/CVT. A mild positive correlation coefficient of 0.285 was observed between ANB and NSL/VER, while a negative correlation coefficient of − 0.299 was observed between ANB and OPT/HOR. Only these two parameters exhibit statistical significances (*P* < 0.05). SNA and SNB was negatively correlated with NSL/CVT (*P* < 0.05) and SNB was inversely correlated with NSL/OPT as well (*P* < 0.05). NSL/NL was negatively correlated with NL/VER, NL/OPT, NL/CVT (*P* < 0.05). There were positive correlations between NSL/ML, FH/ML, and the Y-axis with NSL/VER, NSL/ML was positively correlated with NSL/CVT (*P* < 0.05). In contrast, NP/FH was negatively correlated with NSL/VER (*P* < 0.05). NA/PA has no significant correlation with any of the head and cervical posture variables. However, the correlation coefficients were low in all variables (Table 3).

#### Correlation analysis of variables at the pubertal growth period

ANB was positively correlated with NSL/VER, NL/VER, NSL/OPT, NL/OPT, NSL/CVT, NL/CVT and negatively correlated with CVT/HOR (*P* < 0.05). The correlations between SNA, NSL/NL and all postural parameters were not statistically significant (*P* > 0.05). SNB was negatively correlated with NSL/VER, NL/VER, NSL/OPT, NL/OPT, NSL/CVT, and NL/CVT, while positively correlated with OPT/HOR and CVT/HOR (*P* < 0.05). NSL/ML was positively correlated with NSL/CVT (*P* < 0.05). There were positive correlations between Y-axis, FH/ML and NSL/OPT, NL/OPT, NSL/CVT, NL/CVT (*P* < 0.05). However, there were negative correlations between Y-axis, FH/ML and OPT/HOR, CVT/HOR (*P* < 0.05). A positive relationship between Y-axis and NSL/VER was demonstrated (*P* < 0.05). NA/PA was positively correlated with NSL/VER, NL/VER, NSL/OPT, NL/OPT, NSL/CVT (*P* < 0.05). NP/FH was negatively correlated with NSL/VER, NSL/OPT, NL/OPT, NSL/CVT and NL/CVT (*P* < 0.05). There were positive correlations between NP/FH and OPT/HOR, CVT/HOR (*P* < 0.05). However, the correlation coefficients were generally low for most variables examined (Table 4).

#### Correlation analysis of variables at the post-peak growth periods

ANB was positively correlated with most variables NSL/VER, NL/VER, NL/OPT, NSL/CVT, NL/CVT, and OPT/CVT (*P* < 0.05), but inverse correlations existed in values of SNA, SNB and most craniocervical posture parameters, showing low to moderate coefficients (*P* < 0.05). SNA was negatively correlated with NSL/VER, NSL/OPT, NSL/CVT and positively correlated with OPT/CVT (*P* < 0.05). SNB was negatively correlated with NSL/VER, NL/VER, NSL/OPT, NL/OPT, NSL/CVT and NL/CVT (*P* < 0.05). NSL/NL was positively correlated with NSL/VER, NSL/OPT, NSL/CVT and negatively correlated with OPT/CVT (*P* < 0.05). NSL/ML was positively correlated with NSL/VER (*P* < 0.05). The Y-axis value was positively correlated with NSL/VER, NL/VER, NSL/CVT, NL/CVT (*P* < 0.05). There was no correlation between FH/ML and any head and cervical postural variables (*P* > 0.05). NA/PA was positively correlated with NSL/VER, NL/VER, NSL/OPT, NL/OPT, NSL/CVT, NL/CVT, and OPT/CVT (*P* < 0.05). NP/FH was inversely correlated with NSL/VER, NL/VER, NSL/OPT, NL/OPT, NSL/CVT, NL/CVT (*P* < 0.05) (Table 5).

### A comparison of head and neck postural variables among different skeletal classes within each growth period

Indicators describing the head posture, NSL/VER and NL/VER, were largest in skeletal Class II and smallest in skeletal Class III during the peak and post-peak periods, NSL/VER and NL/VER during the peak growth period, NL/VER during the post-peak growth period have shown statistically significances (*P* < 0.05). NSL/VER and NL/VER was greater in Class II than in Class III at the pre-peak growth period, showing no statistically significances (*P* > 0.05) (Tables 6, 7 and 8).

**Table 6:** Difference analysis among the pre-peak growth period.

Cervical posture variables	Class I	Class II	Class III	*P*
NSL/VER	91.09 ± 11.99	97.58 ± 5.55	91.54 ± 10.82	0.101
NL/VER	88.93 ± 5.22	88.18 ± 5.39	86.65 ± 4.26	0.426
NSL/OPT	100.30 ± 8.49	97.92 ± 10.12	93.82 ± 8.85	0.143
NL/OPT	93.34 ± 7.98	89.39 ± 7.83	82.89 ± 9.99	0.005**
NSL/CVT	101.83 ± 8.74	101.39 ± 9.76	98.25 ± 8.85	0.483
NL/CVT	94.90 ± 8.38	92.72 ± 6.88	87.79 ± 10.09	0.062
OPT/HOR	89.96 ± 6.81	88.44 ± 7.98	95.18 ± 10.48	0.068
CVT/HOR	86.79 ± 8.43	85.82 ± 8.06	90.47 ± 9.76	0.282
OPT/CVT	3.63 ± 3.12	3.87 ± 3.75	4.71 ± 4.52	0.705

*: *P* < 0.05, **: *P* < 0.01.

**Table 7:** Difference analysis among the peak growth period.

Cervical posture variables	Class I	Class II	Class III	*P*
NSL/VER	98.93 ± 5.11	100.34 ± 5.2	95.53 ± 4.26	0.020*
NL/VER	87.7 ± 4.33	90.58 ± 4.94	85.9 ± 5.05	0.022*
NSL/OPT	102.54 ± 8.39	102.83 ± 7.65	94.05 ± 8.7	0.005**
NL/OPT	91.83 ± 8.83	93.58 ± 7.3	83.94 ± 10.74	0.008**
NSL/CVT	105.1 ± 7.88	105.73 ± 6.72	97.4 ± 7.61	0.003**
NL/CVT	94.6 ± 8.53	96 ± 6.34	87.95 ± 9.23	0.014*
OPT/HOR	86.36 ± 7.26	87.27 ± 6.51	91.47 ± 7.66	0.106
CVT/HOR	83.48 ± 6.33	83.91 ± 6.59	88.75 ± 6.97	0.052
OPT/CVT	2.88 ± 2.07	3.36 ± 3.72	2.69 ± 4.01	0.836

*: *P* < 0.05, **: *P* < 0.01.

**Table 8:** Difference analysis among the post-peak growth period.

Cervical posture variables	Class I	Class II	Class III	*P*
NSL/VER	96.91 ± 5.92	99.48 ± 6.52	95.83 ± 6.85	0.244
NL/VER	87.18 ± 4.05	90.24 ± 3.76	85 ± 5.95	0.007**
NSL/OPT	100.29 ± 9.12	102.94 ± 6.48	99 ± 9.84	0.394
NL/OPT	90.77 ± 6.38	92.68 ± 6.31	88.15 ± 9.45	0.220
NSL/CVT	102.37 ± 9	106.36 ± 5.86	100.24 ± 7.88	0.068
NL/CVT	93.56 ± 6.5	96.74 ± 5.64	89.73 ± 8.51	0.019*
OPT/HOR	86.57 ± 6.02	87.09 ± 6.83	87.12 ± 8.14	0.969
CVT/HOR	83.77 ± 5.9	82.61 ± 5.89	85.02 ± 6.81	0.531
OPT/CVT	2.79 ± 2.05	4.43 ± 2.49	2.1 ± 4.08	0.072

*: *P* < 0.05, **: *P* < 0.01.

All parameters describing craniocervical angles (NSL/OPT, NL/OPT, NSL/CVT, NL/CVT) were largest in the skeletal Class II and lowest in the skeletal Class III during the peak and post-peak periods, showing statistical significances in the peak periods and NL/CVT variable during the post-peak periods (*P* < 0.05). The above most variables were largest in skeletal Class I and smallest in skeletal Class III during the pre-peak growth period, but only NL/OPT variable showed a significance (*P* < 0.05) (Table 6). The inclination of the cervical spine (OPT/HOR, and CVT/HOR) was greater in skeletal Class III than skeletal Class II in the overall growth periods, but no statistical significances were shown (*P* > 0.05).

The largest cervical curvature was exhibited in skeletal Class III than skeletal Class II in the pre-peak growth period, which is contrary to the results obtained in the peak and post-peak growth periods, exhibiting the smallest in skeletal Class III and the largest in skeletal Class II, though these differences were not statistically significant (*p* > 0.05). However, corresponding results will require further confirmed.

## Discussion

Under normal physiological conditions, the stability of cervical curvature is not achieved until the age of seven^46^. Previous studies^47,48^ have underscored the significance of attached muscles, especially the sternocleidomastoid muscle^49,50^, in providing stability and supporting the posture of the head and neck. Studies have proposed that the convergence of sensory information at the trigeminal nuclei from various structures such as the periodontium, masticatory muscles, jaws, and cervical spine region can influence the neuroanatomical connections between posture and the stomatognathic system^47,51,52^. Thus, adjustments in head and cervical posture are required to attain a personally perceived comfortable position, which compensates for alterations in stomatognathic homeostasis^53^. Moreover, the head and neck posture gradually reorient themselves towards a normal direction through gravity modifications acting on the craniocervical structures^54^. In a study conducted by Kondo et al.^55^, it demonstrated that an interdisciplinary approach involving early occlusal improvement and physiotherapy to establish a harmonious balance between the neck and masticatory muscles was effective in enhancing both facial appearance and posture.

In the present study, no significant gender difference was found in the correlation of craniofacial morphology with craniocervical posture in sagittal skeletal pattern. It can be inferred that these results are independent of gender, which are in agreement with some of the findings in previous studies^25,30^. Although not all groups exhibited statistically significant differences, it’s worth noting that there are more correlated between most craniofacial morphology variables and craniocervical posture during the peak and post-peak growth periods, which indicated an interrelation between craniofacial morphology and craniocervical posture in the development of sagittal skeletal discrepancies. Furthermore, the correlation between malocclusion and craniocervical condition appears to change with growth. These results suggest that maxilla components may have a less significant impact on determining craniocervical posture compared to the mandibular. As opposed to the maxilla, the results indicate a strong relationship to mandibular-related indicators at the peak growth periods, significant differences were observed in the majority of parameters related to the mandible and craniocervical posture, suggesting that the size and position of the mandible are two primary factors strongly associated with head and cervical posture, which is in line with previous studies^56–59^. A significantly higher extension of the head upon the spine was observed. Therefore, these results underscored the significance of clinicians in assisting adolescent patients seeking orthodontic treatment, clinicians should pay attention to the maturity of the cervical spine as indicated in lateral cephalograms, as well as to identify and address any detrimental postural habits to prevent the development of poor posture and malocclusion.

Cervical curvature (OPT/CVT) is an essential determinant for evaluating the posture of the cervical spine. The measurement of OPT/CVT was greatest in the skeletal Class II and lowest in the skeletal Class III both during the growth spurt period and in subsequent stages. The values of OPT/HOR and CVT/HOR were found to be highest in individuals with skeletal Class III malocclusion across all growth stages, demonstrating a tendency that the cervical spine inclined more dorsally in skeletal Class III malocclusion, while the cervical column tilted more forward in skeletal Class II malocclusion, although this difference was not statistically significant, this trend is consistent with other studies^30,33^. These findings suggest a potential relationship between craniofacial morphology and craniocervical posture in patients with sagittal skeletal malocclusion during the pubertal growth period, which may provide valuable insights into the assessment of head and cervical position in the field of orthodontics. Hence, it would be beneficial for future studies to concentrate on adolescents during or after their peak growth period and to further investigate the association between the cervical spine and various craniomaxillofacial indicators within this stage. This could be a key population for preventing the development of this condition and developing a more comprehensive and interdisciplinary treatment approach.

Although the changes in variables are not stable and these could have an impact on the results of the current study, which may be related to the dynamic growth process that children experience, the current findings display a tendency that the cervical spine was more inclined forward in skeletal Class II and flexed in skeletal Class III, and is strongly supported by other studies^30,32^. The craniomaxillofacial region and cervical spine are susceptible to environmental factors, such as mouth breathing, heavy load, significantly impacting on body posture, particularly during the growth spurt stage ^60,61^. Therefore, it also emphasized the need to address any postural changes as early as possible, preferably within the younger age group. This early intervention aims to mitigate growth deformations, minimize the power investment by the body, and optimize mechanical efficiency^62^. These findings could aid the orientation of future research though the results did not provide us with well-support conclusive results. Taking the effect of natural changes in growing patients into consideration, the present study investigated the relationship between craniofacial morphology with the craniocervical position based on the maturity of second through fourth cervical vertebrae, reflecting a more precise age estimation in individuals with sagittal skeletal discrepancies. To accurately evaluate the growth stage of an individual, compared with chronological age, skeletal age^63^ is considered the optimal determinant, which can reflect individual growth and maturity more accurately and efficiently by examinations of CVM and hand-wrist x-ray mostly. Evidences have demonstrated that the assessment of CVM was comparable to hand-wrist analysis in terms of determining skeletal age. Furthermore, lateral cephalometric radiographs are routinely obtained for clinical orthodontic practice avoiding additional x-ray exposure^64–67^.

However, there remain several limitations in the present study. Therefore. it is necessary to emphasize that the caution should be applied in interpreting and promoting findings presented in this study. Firstly, considering that most skeletal class I patients have dental malocclusion problems such as crowding^68^, anterior open bite^69^, deep overjet^70^, and these factors should also be taken into consideration. Due to ethical considerations, for the absence of control group and its limitations as a cross-sectional study regarding growth evaluation, which is insufficient sensitivity to individual variability. Thus, it is recommended to conduct more studies that evaluate a group of subjects in a longitudinal manner with a multi-center approach to address the limitations of the current study design and enhance the understanding of this topic. Secondly, it is important to note that many of these measurements still remain standardization and validation as reliable tools for postural assessment. Therefore, a comprehensive range of parameters should be employed to accurately depict the craniofacial structures associated with deviated craniocervical posture. A study^11^ suggested the utilization of geometric morphometric methods, is a more effective approach compared to conventional cephalometric analysis for visually evaluating variations in the morphology and size among different skeletal classes. It was found that the curve fitting method is a suitable approach for assessing cervical curvature, although its application in clinical practice is challenging due to the requirement of C7 vertebra tracing. D’Attilio et al.^32^ demonstrated that the lower part of the spine was straighter in skeletal Class III subjects compared to those with skeletal Class I and Class II subjects. It is evident that using the angle formed by the OPT and CVT lines alone to describe cervical column curvature is insufficient. Further investigations are needed to identify more relevant indicators of cervical inclination. Additionally, a more comprehensive approach for appraisal and evaluation of the lower part of the spinal column should be taken into account in future studies.

In fact, head and cervical posture appeared to be associated with both the sagittal facial dimension and the vertical development of the face. These patterns have important implications for clinical diagnosis and treatment prognosis in practice, thus, vertical facial patterns should be taken into account in further research. However, other limitation of the study is that the results did not reveal a cause-effect relationship behind the interactions between occlusion and posture. Therefore, it is crucial to conduct more longitudinal studies with a higher level of evidence and extended follow-up periods to further investigate the relationship. However, it is crucial to highlight the significance of assessing craniocervical posture as an integral part of the clinical practice, as it may play a vital role in facilitating a comprehensive orthodontic diagnosis and treatment planning.

## Conclusion

Variables of craniofacial morphology and craniocervical posture are more correlated during the pubertal growth period and later in patients with sagittal skeletal pattern and a more extended head was displayed in the skeletal Class II relationship while a flexed head was exhibited in the skeletal Class III relationship, These findings highlight significant changes in head and cervical posture during periods of rapid growth, which is important to provide clinical decisions for orthodontic treatment and prognosis in the dynamic process of growth. However, taking the limitations of the study into account, further more prospective studies with large samples, good study designs are needed to clearly elucidate this association.

### Supplementary Information

Supplementary Information.

## Data Availability

All data are available in this manuscript and its supplementary material.
