# Supplementary material for: Craniocervical posture in patients with skeletal malocclusion and its correlation with craniofacial morphology during different growth periods
Source: Sci Rep. 2024 Mar 4;14:5280. doi: 10.1038/s41598-024-55840-w (PMC10912775; doi:10.1038/s41598-024-55840-w)
Supplement: Supplementary file 1 — Supplementary Information. [file 41598_2024_55840_MOESM1_ESM.docx]

**Supplementary Information**


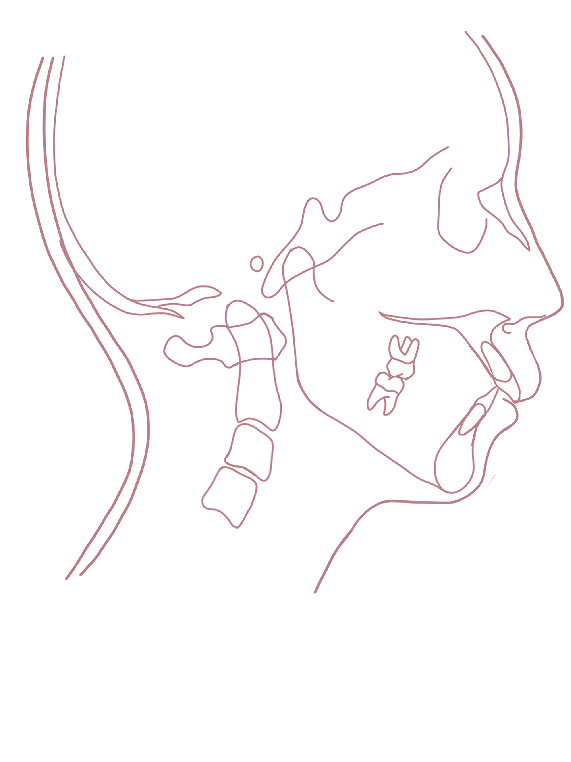


**Figure S1A.** The profiles of skeletal class Ⅱ malocclusion on the Cephalograms


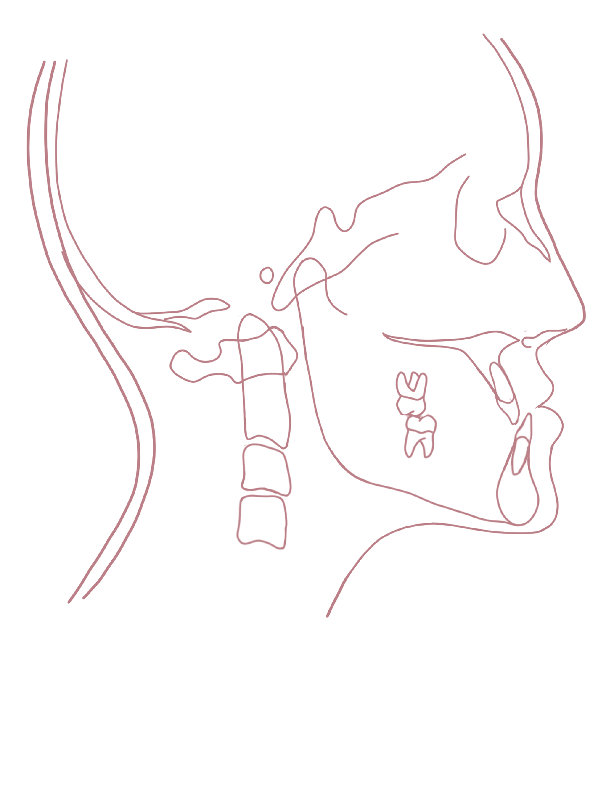


**Figure S1B.** The profiles of skeletal class Ⅲ malocclusion on the Cephalograms

**Table S1.** The definitions of reference points and planes

| **Reference points and planes** | **Description** | **Characterization** |
| --- | --- | --- |
| S | Sella turcica | The midpoint of Sella turcica (1, 2) |
| N | Nasion | the most anterior point on the frontonasal suture (1, 3) |
| A | Point A | the most posterior midline point in the concavity between the anterior nasal spine and the prosthion (3). |
| B | Point B | the most posterior midline point in the concavity of the mandible between the most superior point on the alveolar bone overlying the lower incisors (infradentale) and pogonion (3) |
| Or | Orbitale | the lowest point on the lower margin of the bony orbit midpoint between right and left images (4) |
| Po | Porion | The midpoint on the upper edge of the porus acusticus externus (4) |
| ANS | Anterior Nasal Spine | The most anterior tip of nasal spine (2, 3) |
| PNS | Posterior Nasal Spine | The most posterior point of the maxillary at palatal level (2, 3) |
| P | Pogonion | The most protrusion point of the chin (3) |
| Gn | Gnathion | The mid-point between the Pogonion and Menton points of the bony chin (3) |
| Me | Menton | the lowest point on the of the mandible (3) |
| CV2ip |  | The most postero-inferior point on the corpus of the second cervical vertebra (2, 3) |
| CV2tg |  | The tangent point of odontoid process tangent on the odontoid process of the second cervical vertebra (2, 3) |
| CV4ip |  | The most posterior-inferior point on the corpus of the fourth cervical vertebra (2, 3) |
| NSL | Nasion-Sella Line | Cranial base, line extending from Nasion to Sella (2) |
| NL | Nasal Line | Palatal plane, the line extending from ANS to PNS (2) |
| FH | Frankfort Horizontal | the line extending between Or and Po points (5) |
| NA |  | Line extending from Nasion to A (3, 5) |
| NB |  | Line extending from Nasion to B (3, 5) |
| PA |  | Line extending from Pogonion to A |
| NP |  | Line extending from Nasion to Pogonion. |
| ML | Mandibular line | The tangent to the lower border of the mandible through Me (6) |
| HOR | Horizontal | True horizontal line, the line perpendicular to VER (3, 5) |
| VER | Vertical | True vertical line, plumb line (3, 5) |
| OPT | Odontoid Process Tangent | Posterior tangent to the odontoid process from Cv2ip (1, 3, 5) |
| CVT | Cervical Vertebra Tangent | Posterior tangent to the odontoid process from Cv4ip (1, 3, 5) |

**References:**

1. Solow B, Tallgren A. Head posture and craniofacial morphology. Am J Phys Anthropol. 1976;44(3):417-35.

2. Pachi F, Turla R, Checchi AP. Head posture and lower arch dental crowding. Angle Orthod. 2009;79(5):873-9.

3. Garg TT, Khanna R, Pratap R, Maurya K, Srivastava SL. Is Head Posture and Malocclusion Related? J Contemp Orthod. 2019;3(3):38-47.

4. D'Attilio M, Caputi S, Epifania E, Festa F, Tecco S. Evaluation of cervical posture of children in skeletal class I, II, and III. Cranio. 2005;23(3):219-28.

5. Hedayati Z, Paknahad M, Zorriasatine F. Comparison of natural head position in different anteroposterior malocclusions. J Dent (Tehran). 2013;10(3):210-20.

6. Liu Y, Sun X, Chen Y, Hu M, Hou X, Liu C. Relationships of sagittal skeletal discrepancy, natural head position, and craniocervical posture in young Chinese children. Cranio. 2016;34(3):155-62.
